# Melt Conveying in Single-Screw Extruders: Modeling and Simulation

**DOI:** 10.3390/polym14050875

**Published:** 2022-02-23

**Authors:** Christian Marschik, Wolfgang Roland, Tim A. Osswald

**Affiliations:** 1 Competence Center CHASE GmbH, Altenbergerstrasse 69, 4040 Linz, Austria; 2 Institute of Polymer Processing and Digital Transformation, Johannes Kepler University Linz, 4040 Linz, Austria; wolfgang.roland@jku.at; 3 Polymer Engineering Center, Department of Mechanical Engineering, University of Wisconsin-Madison, Madison, WI 53715, USA; tosswald@wisc.edu

**Keywords:** modeling and simulation, melt conveying, single-screw extruders, polymer processing

## Abstract

Numerous analyses have modeled the flow of polymer melts in the melt-conveying zones of single-screw extruders. While initial studies mainly provided exact analytical results for combined drag and pressure flows of Newtonian fluids, more recently developed, numerical methods seek to deepen the understanding of more realistic flow situations that include shear-thinning and non-isothermal effects. With the advent of more powerful computers, considerable progress has been made in the modeling and simulation of polymer melt flows in single-screw extruders. This work reviews the historical developments from a methodological point of view, including (1) exact analytical, (2) numerical, and (3) approximate methods. Special attention is paid to the mathematical models used in each case, including both governing flow equations and boundary conditions. In addition, the literature on leakage flow and curved-channel systems is revisited.

## 1. Introduction

Over the past century, the technical progress of extruders in polymer processing has gone hand in hand with extensive theoretical and experimental research summarized in numerous books [1,2,3,4,5,6,7,8,9,10,11,12,13,14,15,16,17,18] and reviews [19,20,21,22,23,24]. Recently, Wilczyńnski et al. [25] provided a comprehensive literature survey of the modeling and simulation of single- and twin-screw extruders, placing special emphasis on global modeling aspects. Similarly, Hyvärinen et al. [26] reviewed the existing extrusion theories, focusing on the processing behavior of composite materials.

**Figure 1:**
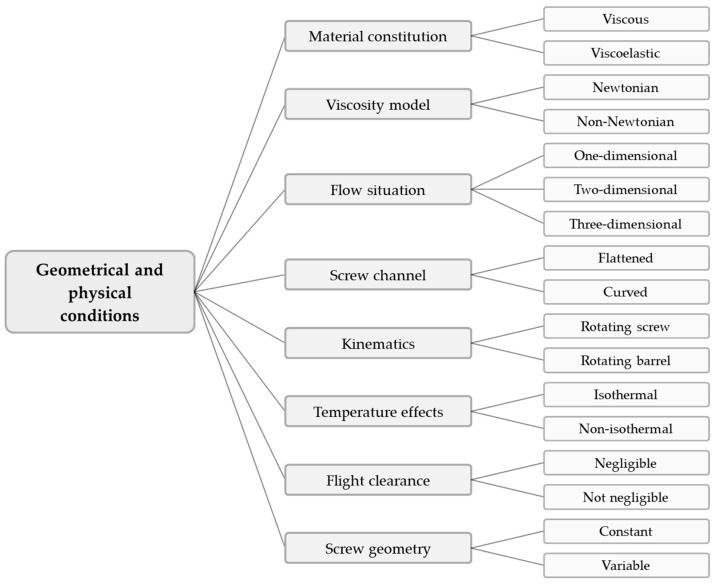
Model categorization A: Classification of melt-conveying models according to the geometric and physical conditions under consideration.

**Figure 2:**
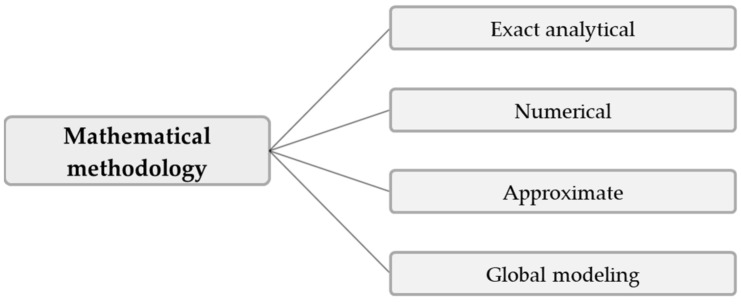
Model categorization B: Classification of melt-conveying models according to the mathematical methodology applied.

Rather than tracing the developments in the modeling and simulation of polymer extrusion in general, the purpose of this review is to specifically address the main progress in the mathematical analysis of melt conveying in single-screw extruders. This functional step has received significant attention in the extrusion literature since the first theories describing screw viscosity pumps were formulated in the 1920s. With the continuous development of more advanced computers, numerous melt-conveying models of increasing complexity and accuracy have been presented. These can be classified on the basis of (a) the geometrical and physical conditions under consideration, or (b) the underlying mathematical methodology, as illustrated in Figure 1 and Figure 2, respectively.

This work reviews the existing theories from a methodological viewpoint, including (1) exact analytical, (2) numerical, and (3) approximate approaches. For a comprehensive review of global extrusion modeling, see Wilczyńnski’s work [25]. In the first part of this paper, we revisit the fundamentals of the modeling of polymer melt flows in single-screw extruders. We then compare the various methods and summarize the corresponding theories. For convenience, the literature on leakage flow and curved-channel systems is presented in additional sections.

## 2. Modeling Fundamentals

### 2.1. Screw Geometry

**Figure 3:**
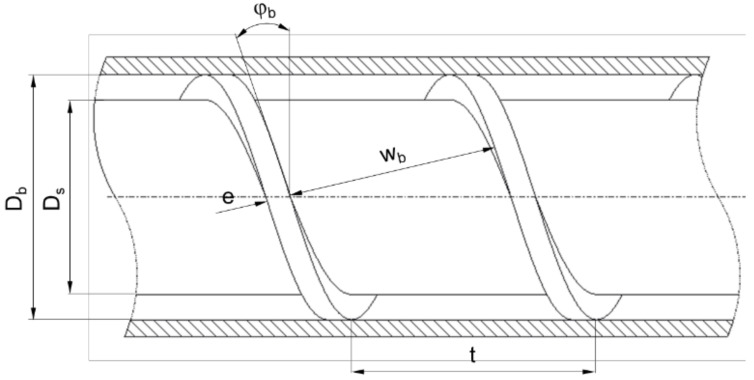
Helical screw channel.

Figure 3 presents the basic geometry of a single-flighted extruder screw section. The most important parameters are the barrel diameter $D_{b}$, the channel depth h, the screw pitch t, and the flight width e. The screw core diameter $D_{s}$ can be calculated according to Equation (1). The pitch angle and the channel width are functions of the radial screw position. At the barrel diameter, the parameters $\varphi_{b}$ and $w_{b}$ are obtained from Equations (2) and (3), respectively.

Several coordinate systems can be used to describe the flow along the helical screw channel. Calculations in helical coordinates have only been presented by a limited number of authors [27,28,29,30,31,32,33,34,35,36]; the main reason is that the governing flow equations in these coordinates are not very well established (see Section 7).

(1) $$D_{s}=D_{b}-2\;h\;$$

(2) $$tan\left(\varphi_{b}\right)=\frac{t}{D_{b}\;\pi }$$

(3) $$w_{b}=\frac{D_{b}\;\pi \;sin\left(\varphi_{b}\right)}{i}-e\;$$

**Figure 4:**
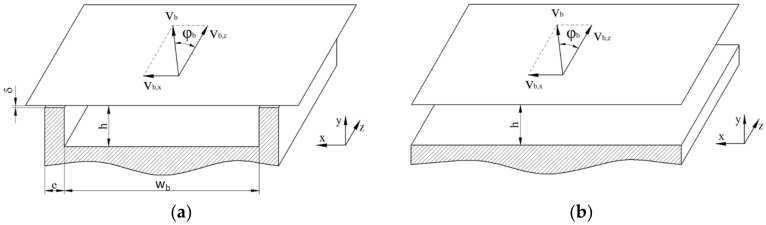
Flat-plate model of the helical screw channel (**a**). For shallow screw channels with $h/w_{b}<0.1$, the effect of the screw flights can be ignored (**b**).

To avoid the helical reference frame, the screw channel is often conceptually unwound and laid out on a flat plane. In most cases, the screw is unwrapped at the radial position of the barrel. The result of this approximation—widely referred to as a flat-plate model—is a straight rectangular screw channel covered by a flat plate, which represents the barrel surface (Figure 4a). Note that, when using the flat-plate model, the dependency of geometric parameters on the radial position is omitted. When unwinding the screw at the radial position of the barrel, the cross-section of the flow channel is hence overestimated. Calculations based on an average channel width were presented in [6].

The curvature of the screw being ignored, the flow can be described by a Cartesian coordinate system with x, y, and z denoting the cross-, up-, and down-channel directions, respectively. The error introduced by unwinding the screw is small for screw sections with small $h/D_{b}$, and becomes more significant for deeper channels [37]. In addition, for shallow channels with $h/w_{b}<0.1$, the influence of the screw flights can be ignored [7], which gives rise to an infinitely wide screw channel, as shown in Figure 4b.

Traditionally, the flat-plate model has been used in combination with kinematic reversal. This entails that the screw is considered to be stationary and the barrel surface to be moving at circumferential speed $v_{b}$ at an angle of $\varphi_{b}$ with respect to the down-channel direction. Analyzing the flow from a reference frame attached to the screw, the barrel velocity can be decomposed into components in the cross- and down-channel directions, $v_{b,x}$ and $v_{b,z}$, respectively:

(4) $$v_{b}=D_{b}\;\pi \;N\;$$

(5) $$v_{b,z}=v_{b}\;cos\left(\varphi_{b}\right){\;\;\;\;\;\;\;\;\;\;v}_{b,x}=v_{b}\;sin\left(\varphi_{b}\right)$$

In the early 1990s, several studies proposed an alternative kinematic approach that considers the screw as the rotating component in the flat-plate system [38,39,40,41,42]. A thorough textbook on screw rotation modeling was presented by Campbell and Spalding [6].

Rauwendaal et al. [43,44] critically reappraised the validity of the two kinematic approaches in combination with the flat-plate model. Assuming the flow of a Newtonian fluid, the methods were shown to differ only slightly for the normal range of channel depth values ($h/D_{b}<0.1)$. For the case of the channel depth being large in relation to the barrel diameter ($h/D_{b}>0.2)$, it was demonstrated that the inability of the flat-plate model to properly account for the screw curvature causes errors in both theories; these were more pronounced when the moving-screw assumption was applied.

### 2.2. Conservation Equations

The differential equations governing all types of flow are the conservation equations of mass, momentum, and energy [45]:

(6) $$\frac{\partial \rho }{\partial t}+\nabla \cdot \left(\rho \;v\right)=0$$

(7) $$\frac{\partial }{\partial t}\left(\rho \;v\right)+\nabla \cdot \left(\rho \;\mathrm{vv}\right)=-\nabla p+\nabla \cdot \tau +\rho \;g$$

(8) $$\rho c_{v}\left(\frac{\partial T}{\partial t}+\nabla \cdot \left(T\;v\right)\right)=\nabla \cdot \left(\lambda \;\nabla T\right)-T{\left(\frac{\partial p}{\partial T}\right)}_{\rho }\left(\nabla \cdot v\right)+\tau :L$$

In the past, theoretical analyses of melt conveying in single-screw extruders were commonly based on various modeling assumptions. Typically, the flow is considered to be in a steady state; that is, time-dependent effects due to, e.g., transient changes in extrusion conditions, are ignored. Furthermore, the polymer melt is often assumed to be incompressible, which implies that the density is locally constant. In addition, the flow of polymer melts in metering channels is usually laminar. Due to the high viscosity of polymer melts and the low fluid velocities in the channel, the flow is predominantly governed by viscous rather than inertial forces. A dimensionless parameter representing the ratio of inertial to viscous forces is the Reynolds number (Re). For a power-law fluid, this parameter is defined by Equation (9), where L is a characteristic length and v a characteristic fluid velocity [46]:

(9) $$Re=\frac{\rho \;L^{n}}{K\;v^{n-2}}$$

For most extrusion conditions, Re≪1; thus, the flow can be reduced to Stokes flow. In combination with the aforementioned simplifications, the flow can be described by Equations (10)–(12):

(10) ∇·v=0

(11) ∇p=∇·τ

(12) $$\rho c_{v}\;\left(v\cdot \left(\nabla T\right)\right)=\nabla \cdot \left(\lambda \;\nabla T\right)+\tau :L$$

### 2.3. Constitutive Equations

A complete description of the governing flow equations requires definition of the thermodynamic and rheological properties of the fluid. In general, polymer melts exhibit shear-thinning and viscoelastic effects. In the analysis of single-screw extruders, it is widely accepted that elastic time effects play only a minor role, as the polymer melt passing through the screw channel is typically subjected to large deformation rates for relatively long periods. For this reason, the polymer melt is usually treated as an inelastic viscous fluid characterized by the generalized Newtonian fluid constitutive relation given in Equation (13). To date, viscoelastic effects, which can be described by many complex constitutive equations, have received relatively little attention in extrusion modeling. Published work is available for the flow through extrusion dies [47,48,49] and melt-conveying zones [36,50,51,52]. The main reason for the scarcity of work on viscoelastic flow analysis is the high computational power required for these types of flow.

(13) τ=2 η D

(14) $$D=\frac{1}{2}\left(L+L^{T}\right)$$

(15) L=∇v

A simple viscosity model for polymer melts is the power law (Equation (16)) according to Ostwald and de Waele [53,54]. On a log–log scale, the power law is a linear function. Since slope and intercept depend on the shear rate at which the mathematical fit of the experimental data is performed, the model works well only within a specific range of shear rates. To approximate the viscosity behavior of polymer melts in the terminal and shear-thinning regimes, numerous models of increased complexity are available in the literature [4]. Since most melt-conveying models that include shear-thinning flow behavior are based on the power law, detailed descriptions of other approaches are avoided. For multidimensional flows, the magnitude of the shear rate can be calculated according to Equation (17):

(16) $$\eta =K\;{\left|\dot{\gamma }\right|}^{n-1}a_{t}$$

(17) $$\left|\dot{\gamma }\right|=\sqrt{2\left(D:D\right)}$$

The temperature sensitivity of the viscosity can be described by the temperature-shift factor $a_{t}$, which is frequently approximated by a simplified Arrhenius-type relationship (Equation (18)). Note that $a_{t}=1$ for isothermal flows. The effect of pressure on viscosity, in contrast, is usually ignored.

(18) $$a_{t}=exp\left[-\alpha \left(T-T_{0}\right)\right]$$

### 2.4. Fully Developed Flows

A widely used simplification in the analysis of flow in single-screw extruders is the assumption of a hydrodynamically fully developed flow. When using the flat-plate model (Figure 4a), Equations (10) and (11) can be further simplified by introducing the lubrication approximation [4]. According to this theory, the velocities in the down-channel direction may be regarded as being fully developed. This means that the velocities $v_{x}$, $v_{y}$, and $v_{z}$ are functions of x and y only. Consequently, the continuity and momentum equations can be reduced to Equations (19)–(22), whose stress components $\tau_{ij}$ are obtained from Equations (13)–(15). Furthermore, the magnitude of the shear rate can be described by Equation (23).

(19) $$\frac{\partial v_{x}}{\partial x}+\frac{\partial v_{y}}{\partial y}=0$$

(20) $$\frac{\partial p}{\partial x}=\frac{\partial \tau_{xx}}{\partial x}+\frac{\partial \tau_{xy}}{\partial y}=\frac{\partial }{\partial x}\left(2\;\eta \;\frac{\partial v_{x}}{\partial x}\right)+\frac{\partial }{\partial y}\left[\eta \left(\frac{\partial v_{x}}{\partial y}+\frac{\partial v_{y}}{\partial x}\right)\right]$$

(21) $$\frac{\partial p}{\partial y}=\frac{\partial \tau_{yx}}{\partial x}+\frac{\partial \tau_{yy}}{\partial y}=\frac{\partial }{\partial x}\left[\eta \left(\frac{\partial v_{x}}{\partial y}+\frac{\partial v_{y}}{\partial x}\right)\right]+\frac{\partial }{\partial y}\left(2\;\eta \;\frac{\partial v_{y}}{\partial y}\right)$$

(22) $$\frac{\partial p}{\partial z}=\frac{\partial \tau_{zx}}{\partial x}+\frac{\partial \tau_{zy}}{\partial y}=\frac{\partial }{\partial x}\left(\eta \;\frac{\partial v_{z}}{\partial x}\right)+\frac{\partial }{\partial y}\left(\eta \;\frac{\partial v_{z}}{\partial y}\right)$$

(23) $$\left|\dot{\gamma }\right|={\left[2{\left(\frac{\partial v_{x}}{\partial x}\right)}^{2}+{\left(\frac{\partial v_{x}}{\partial y}+\frac{\partial v_{y}}{\partial x}\right)}^{2}+2{\left(\frac{\partial v_{y}}{\partial y}\right)}^{2}+{\left(\frac{\partial v_{z}}{\partial x}\right)}^{2}+{\left(\frac{\partial v_{z}}{\partial y}\right)}^{2}\right]}^{0.5}$$

A number of analyses additionally apply the lubrication approximation to the temperature distribution in the z-direction. If a thermally fully developed flow is assumed, the energy equation can be reduced to Equation (24). This means that the heat generated internally must be conducted away entirely through the screw and barrel surfaces, and that there is no convective heat transfer in the down-channel direction. As the thermal conductivity of polymers is generally low, this simplification represents only a rare case of extruder operation. Hunter and Zienkiewicz [55] demonstrated the effects of temperature variations across lubricating films.

(24) $$\rho c_{v}\left(v_{x}\frac{\partial T}{\partial x}+v_{y}\frac{\partial T}{\partial y}\right)=\lambda \left(\frac{\partial^{2}T}{\partial x^{2}}+\frac{\partial^{2}T}{\partial y^{2}}\right)+\tau_{xx}\frac{\partial v_{x}}{\partial x}+\tau_{yy}\frac{\partial v_{y}}{\partial y}+\tau_{xy}\left(\frac{\partial v_{x}}{\partial y}+\frac{\partial v_{y}}{\partial x}\right)+\tau_{zx}\frac{\partial v_{z}}{\partial x}+\tau_{yz}\frac{\partial v_{z}}{\partial y}$$

The three-dimensional model can be further simplified by assuming the channel aspect ratio $h/w_{b}$ to be large, as shown in Figure 4b. The screw flights being ignored, the flow field is two-dimensional, with two non-zero-velocity components, $v_{x}$ and $v_{z},$ which are independent of x. In this case, the continuity equation is implicitly fulfilled, and the flow is governed by Equations (25)–(28). The magnitude of the shear rate can be obtained from Equation (29).

(25) $$\frac{\partial p}{\partial x}=\frac{d\tau_{xy}}{dy}=\frac{d}{dy}\left(\eta \;\frac{dv_{x}}{dy}\right)$$

(26) $$\frac{\partial p}{\partial y}=0$$

(27) $$\frac{\partial p}{\partial z}=\frac{d\tau_{zy}}{dy}=\frac{d}{dy}\left(\eta \;\frac{dv_{z}}{dy}\right)$$

(28) $$\lambda \frac{d^{2}T}{dy^{2}}=-\tau_{xy}\frac{dv_{x}}{dy}-\tau_{yz}\frac{dv_{z}}{dy}$$

(29) $$\left|\dot{\gamma }\right|={\left[{\left(\frac{dv_{x}}{dy}\right)}^{2}+{\left(\frac{dv_{z}}{dy}\right)}^{2}\right]}^{0.5}$$

For the one-dimensional down-channel flow, the momentum equation is given by Equation (27). Moreover, the energy equation and the magnitude of the shear rate result from Equations (30) and (31), respectively.

(30) $$\lambda \frac{d^{2}T}{dy^{2}}=-\tau_{yz}\frac{dv_{z}}{dy}$$

(31) $$\left|\dot{\gamma }\right|=\left(\frac{dv_{z}}{dy}\right)$$

### 2.5. Developing Flows

While the assumption of a hydrodynamically fully developed flow is usually justified (Re≪1), some studies have emphasized the dominant effect of convective heat transport in the energy equation at higher Péclet numbers ($Pe\approx 10^{4}$) [14,56]. This dimensionless parameter describes the ratio of convective to diffusive transport rate, and is defined by Equation (32):

(32) $$Pe=\frac{\rho \;L\;v\;c_{p}}{\lambda }$$

Especially in large extruders, a thermally fully developed flow will not necessarily be achieved even at the exit of the processing machine [57]. In a hydrodynamically fully developed flow, convective heat transfer can be considered by Equations (33) and (34) for three- and two-dimensional flows, respectively:

(33) $$\rho c_{p}\left(v_{x}\frac{\partial T}{\partial x}+v_{y}\frac{\partial T}{\partial y}+v_{z}\frac{\partial T}{\partial z}\right)=\lambda \left(\frac{\partial^{2}T}{\partial x^{2}}+\frac{\partial^{2}T}{\partial y^{2}}\right)+\tau_{xx}\frac{\partial v_{x}}{\partial x}+\tau_{yy}\frac{\partial v_{y}}{\partial y}+\tau_{xy}\left(\frac{\partial v_{x}}{\partial y}+\frac{\partial v_{y}}{\partial x}\right)+\tau_{zx}\frac{\partial v_{z}}{\partial x}+\tau_{yz}\frac{\partial v_{z}}{\partial y}$$

(34) $$\rho c_{p}v_{z}\frac{\partial T}{\partial z}=\lambda \frac{\partial^{2}T}{\partial y^{2}}+\tau_{yx}\frac{\partial v_{x}}{\partial y}+\tau_{yz}\frac{\partial v_{z}}{\partial y}$$

### 2.6. Boundary Conditions and Mathematical Constraints

Most melt-conveying models in the literature were developed under the no-slip condition, which results in the following conditions in the case of the flat-plate model with reversed kinematics (Figure 4a):

(35) $$v_{x}\left(x=0\right)=0\;\;\;\;\;\;\;\;\;\;v_{x}\left(x=w_{b}\right)=0\;\;\;\;\;\;\;\;\;\;\;v_{x}\left(y=0\right)=0\;\;\;\;\;\;\;\;\;\;\;v_{x}\left(y=h\right)=v_{b,x}$$

(36) $$v_{y}\left(x=0\right)=0\;\;\;\;\;\;\;\;\;\;v_{y}\left(x=w_{b}\right)=0\;\;\;\;\;\;\;\;\;\;v_{y}\left(y=0\right)=0\;\;\;\;\;\;\;\;\;\;\;v_{y}\left(y=h\right)=0\;\;\;\;$$

(37) $$v_{z}\left(x=0\right)=0\;\;\;\;\;\;\;\;\;\;v_{z}\left(x=w_{b}\right)=0\;\;\;\;\;\;\;\;\;\;\;\;v_{z}\left(y=0\right)=0\;\;\;\;\;\;\;\;\;\;\;v_{z}\left(y=h\right)=v_{b,z}$$

**Figure 5:**
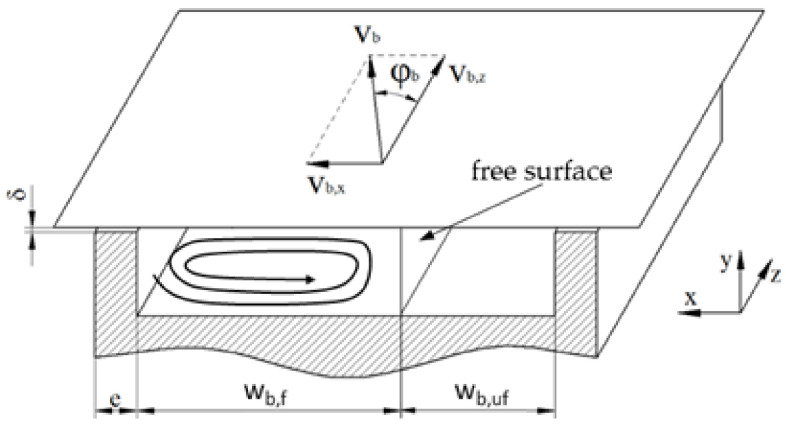
Partially filled screw channel. The widths of the filled and unfilled channel sections are $w_{b,f}$ and $w_{b,uf}$, respectively.

In a partially filled screw channel, as shown in Figure 5, the flow field exhibits a free surface rather than being constrained by the trailing flight, which requires the shear stresses to be zero.

(38) $$v_{x}\left(x=w_{b,uf}\right)=0\;\;\;\;\;\;\;\;\;\;\;\;\;\;\;{\left.\frac{\partial v_{y}}{\partial x}\right|}_{x=w_{b,uf}}=0\;\;\;\;\;\;\;\;\;\;\;\;\;\;\;{\left.\frac{\partial v_{z}}{\partial x}\right|}_{x=w_{b,uf}}=0$$

It is widely known that, under particular conditions, some materials, such as filled polymers, elastomers, and polyvinyl chloride, exhibit wall slippage—that is, a relative velocity between the fluid velocity at the wall and the wall velocity. Numerous studies have used a variety of slip conditions to account for wall slippage [58,59,60,61,62,63,64,65,66].

Prediction of the temperature distribution in the screw channel additionally requires the use of thermal boundary conditions, which may specify either the absolute temperature of the screw and barrel surfaces or the heat fluxes normal to the walls. A critical discussion of the validity of these conditions was given in [21].

(39) $$T\left(y=h\right)=T_{b}\left(z\right)$$

(40) $$T\left(x=0\right)=T_{s}\left(z\right)\;\;\;\;\;\;\;\;\;\;\;\;\;\;\;\;\;\;T\left(x=w\right)=T_{s}\left(z\right)\;\;\;\;\;\;\;\;\;\;\;\;\;\;\;\;\;\;T\left(y=0\right)=T_{s}\left(z\right)$$

(41) $${\left.\frac{\partial T}{\partial x}\right|}_{x=0}=0\;\;\;\;\;\;\;\;\;\;\;\;\;\;\;\;\;\;{\left.\frac{\partial T}{\partial x}\right|}_{x=w}=0\;\;\;\;\;\;\;\;\;\;\;\;\;\;\;\;\;\;{\left.\frac{\partial T}{\partial y}\right|}_{y=0}=0$$

Frequently, the effect of the flight clearance is ignored in flow analyses of infinitely wide screw channels, which entails that the cross-channel net flow is zero.

(42) $$\overset{h}{\underset{0}{\int }}v_{x}dy=0$$

## 3. Exact Analytical Approaches

The first theoretical analyses of polymer melt flows in single-screw extruders dealt with Newtonian fluids with temperature-independent viscosity. Assuming the viscosity to be constant uncouples the energy equation from the continuity and momentum equations. The mathematical problem was further simplified by assuming the flow to be fully developed, which allowed the down- and cross-channel flows to be investigated independently.

Most studies that published closed-form analytical solutions for the flow in single-screw extruders applied the flat-plate approximation (Figure 4). For Newtonian fluids, the material transport along the channel due to the relative movement between barrel and screw and the pressure gradient caused by the restrictive effect of the die is independent of the circulatory transverse flow. The net rate of discharge is therefore governed solely by the velocity distribution in the down-channel direction. Assuming a laminar steady-state flow of an incompressible fluid, the velocity field follows Equation (22). This elliptic partial differential equation is often referred to as Poisson’s equation.

The fluid velocity varies over the depth and lateral position of the screw channel. Closed-form analytical solutions to this non-homogeneous differential equation were derived for various boundary conditions by using the method of separation of variables. To further simplify the mathematical problem, several authors assumed the flow to take place in the y−z mid-plane between the screw flights. In this case, the momentum equation is reduced to Equation (27). Table 1 provides an overview of mathematical models developed for the flow of Newtonian fluids in metering channels.

### 3.1. Flow Pattern and Pumping Capability

The relationship between the flow rate and pressure gradient of viscous fluid flows has been of interest since long before the invention of screw viscosity pumps or plasticating single-screw extruders. In 1868, Boussinesq [67] addressed the topic for a pure pressure flow in narrow tubes. The first mathematical model of screw-type viscous pumps was published anonymously in 1922 [68], and later extended by Rowell and Finlayson in 1928 [69], who investigated an isothermal flow of a Newtonian fluid. To simplify the helical flow geometry, the screw pump was represented by a straight channel filled with an incompressible viscous fluid and covered by a plate maintained at a steady motion. The studies solved the combined drag and pressure flow for screw channels of both infinite and finite widths, as described by the models in Table 1 (1D_a and 1D_b, respectively).

In the 1950s, these initial attempts to model screw pumps were rediscovered and adapted to the characteristics of screw extrusion. It is little known that Maillefer [70] derived solutions for the down-channel velocity profile. At around the same time, scientists at the Polychemicals Department (E. I. du Pont de Nemours and Company, Wilmington, DE, USA) extended the existing extrusion theory in several publications [19,71,72,73,74,75]. The groundwork was laid by Carley and Strub [19], who reviewed the historical developments in the analysis of extrusion flow. Later, Meskat [76] compared the existing solutions for the combined drag and pressure flow, and demonstrated their equivalence. On the basis of the exact theory, the down-channel velocity distribution subject to the equations and boundary conditions in Table 1 (1D_b) can be described by [1]:

(43) $$v_{z}\left(x,y\right)=\frac{v_{z}}{v_{b,z}}=v_{z,d}-v_{z,p},$$

where the velocity profiles resulting from drag and pressure are given as follows:

(44) $$v_{z,d}=\frac{4\;}{\pi }\overset{\infty }{\underset{n=1,3,5}{\sum }}\frac{1}{n}\frac{sinh\left(\frac{n\;\pi \;y}{w_{b}}\right)}{sinh\left(\frac{n\;\pi \;h}{w_{b}}\right)}sin\left(\frac{n\;\pi \;x}{w_{b}}\right),$$

(45) $$v_{z,p}=-\frac{h^{2}}{2\eta v_{b,z}}\frac{\partial p}{\partial z}\left(\frac{y^{2}}{h^{2}}-\frac{y}{h}+\frac{8}{\pi^{3}}\overset{\infty }{\underset{n=1,3,5}{\sum }}\frac{1}{n^{3}}\frac{cosh\left(\frac{n\pi w_{b}}{h}\left(\frac{x}{w_{b}}-\frac{1}{2}\right)\right)}{cosh\left(\frac{n\;\pi \;w_{b}}{2h}\right)}sin\left(\frac{n\;\pi \;y}{h}\right)\right)$$

The net flow rate is obtained by integrating the velocity distribution over the free cross-sectional area [1]:

(46) $$\dot{V}={\dot{V}}_{d}-{\dot{V}}_{p}=\frac{i\;w_{b}\;h\;v_{b,z}}{2}F_{d}+\frac{i\;w_{b}\;h^{3}}{12\;\eta }\frac{\partial p}{\partial z}F_{p},$$

(47) $$F_{d}=\frac{16\;w_{b}}{\pi^{3}\;h}\overset{\infty }{\underset{n=1,3,5}{\sum }}\frac{1}{n^{3}}tanh\left(\frac{n\;\pi \;h}{2\;w_{b}}\right),$$

(48) $$F_{p}=1-\frac{192\;h\;}{\pi^{5}\;w_{b}}\overset{\infty }{\underset{n=1,3,5}{\sum }}\frac{1}{n^{5}}tanh\left(\frac{n\;\pi \;w_{b}}{2\;h}\right)$$

**Figure 6:**
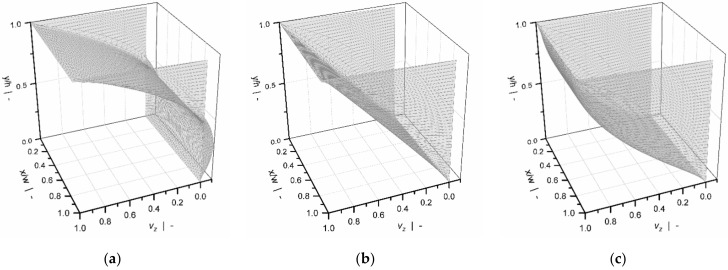
Dimensionless down-channel velocity distribution for a pressure-generating screw section (**a**), a pressure-neutral screw section (**b**), and a pressure-consuming screw section (**c**).

The solution consists of two independent terms: (1) a drag flow, and (2) a pressure flow. For Newtonian fluids, the net flow rate results from linear superposition of the flow components, where $F_{d}$ and $F_{p}$ are the shape factors for the drag and pressure flows, respectively. These parameters, which depend on the aspect ratio of the channel $h/w_{b}$, represent the distortion of the flow field in the vicinity of the flight. Figure 6 illustrates down-channel velocity profiles for a pressure-generating, a pressure-neutral, and a pressure-consuming flow. In all situations, the surfaces of the screw flights retard the motion of the fluid.

To additionally cover the situation in which the screw channel is only partially filled, Squires [77] solved Equation (22) by ignoring the pressure gradient and using the boundary conditions in (38); he expressed the shape factor for the drag flow as a function of the degree of filling f, where $w_{b,f}=w_{b}\;f$.

(49) $$F_{d,f}=\frac{32\;w_{b}\;f^{2}}{\pi^{3}\;h}\overset{\infty }{\underset{n=1,3,5}{\sum }}\frac{1}{n^{3}}tanh\left(\frac{n\;\pi \;h}{4\;f\;w_{b}}\right)$$

A simplified approach to predicting the flow in shallow screw channels ($h/w_{b}<0.1$) was proposed by Carley et al. [71]; omitting the effect of the screw flights, they solved the combined drag and pressure flow between parallel plates in relative motion. This type of flow is governed by the mathematical formulation in Table 1 (1D_b). In their simplified theory, $F_{d}=F_{p}=1$. The down-channel velocity profile is obtained from Equation (50):

(50) $$v_{z}\left(y\right)=\frac{v_{z}}{v_{b,z}}=\frac{y}{h}-\frac{1}{2\;v_{b,z}\;\eta }\;\frac{dp}{dz}\left(y\;h-y^{2}\right)$$

The first analysis of transverse flow of a Newtonian fluid was published by Mohr et al. [74]. In this simplified theory, the transverse velocity profile in the channel center is described by the equations and boundary conditions in Table 1 (1D_c). In the absence of leakage flow, the cross-channel velocity profile is described by Equation (51):

(51) $$v_{x}\left(y\right)=\frac{v_{x}}{v_{b,x}}=\frac{y}{h}\left(3\;\frac{y}{h}-2\right)$$

Many years later, Kaufmann [78] developed a closed-form analytical solution for the recirculating transverse flow. To avoid singularities in the top corners of the channel, he omitted the velocity gradients $\partial v_{x}/\partial x$ and $\partial v_{y}/\partial x$ in his model, which is defined in Table 1 (1D_d). In fluid mechanics, this type of flow is widely known as the lid-driven cavity problem, which has been thoroughly studied [79,80,81]. An alternative approach to describing the cross-channel flow in partially filled screw channels was proposed by Marschik et al. [82].

Even for Newtonian fluids, a full description of the three-dimensional helical flow pattern is a complex task. To gain a deeper understanding of the nature of the flows, Mohr and Mallouk [75] combined the velocity profiles in Equations (50) and (51) to describe the axial velocity profile in the middle of the channel.

### 3.2. Dissipation and Power Consumption

The first attempt to mathematically describe the energy efficiency of a combined drag and pressure flow was presented by Rowell and Finlayson in 1928 [69]. Later, Mallouk and McKelvey [72] addressed the power requirements of melt extruders. On the basis of the simplified theory as initially proposed by Carley et al. [71], they described the total power as the sum of the power consumed in the screw channel and that dissipated in the flight clearance. This theory was refined by Mohr and Mallouk [75] and Gore and McKelvey [83], who included the previously ignored transverse flow. McKelvey [73] introduced calculation of the adiabatic melt temperature development for a temperature-dependent Newtonian fluid, leading to a logarithmic melt temperature increase. Campbell et al. [84] investigated the viscous dissipation rate of Newtonian fluids based on screw-rotation and barrel-rotation theories.

The viscous dissipation rate (Equation (50)) is part of the energy equation, and for polymer melt flows the main causes of melt temperature increase are their high viscosity and low thermal heat conductivity.

(52) $${\dot{q}}_{Diss}=\tau :L$$

Evaluation of the volume-specific viscous dissipation rate in the screw channel requires the velocity profiles in Equations (50) and (51) to be differentiated and the results to be applied to Equation (52). Integration over the cross-channel area yields the viscous dissipation rate per unit of down-channel length.

(53) $${\dot{Q}}_{Diss}=w\left[\eta \frac{v_{b,z}^{2}}{h}+\frac{h^{3}}{12\;\eta }{\left(\frac{\partial p}{\partial z}\right)}^{2}+4\;\eta \;\tan{\left(\varphi_{b}\right)}^{2}\;\frac{v_{b,z}^{2}}{h}\right]$$

The first term represents the viscous dissipation caused by the drag flow, the middle term represents the pressure flow, and the last term represents the cross-channel flow component. There are two possible approaches to determining the required drive power, which lead to the same result. The drive power is the sum of the viscous dissipation rate and pumping power, and can also be computed as the product of shear stress at the moving wall and its velocity.

(54) $$P_{Drive}={\dot{Q}}_{Diss}+\frac{\partial p}{\partial z}\;\dot{V}=w_{b}\;\left({\left.\tau_{yz}\right|}_{y=h}\;v_{b,z}+{\left.\tau_{xy}\right|}_{y=h}\;v_{b,x}\right)$$

The drive power for the simplified Newtonian flow theory results in:

(55) $$P_{Drive}=w\left[\eta \frac{v_{b,z}^{2}}{h}+\frac{h\;v_{b,z}}{2}\frac{\partial p}{\partial z}+4\;\eta \;\tan{\left(\varphi_{b}\right)}^{2}\;\frac{v_{b,z}^{2}}{h}\right]$$

## 4. Numerical Approaches

The early theories of the melt-conveying zone in single-screw extruders analyzed the flow of Newtonian fluids in screw channels of finite or infinite width. To gain more insight into the conveying behavior of extruder screws, several researchers relaxed the constant-viscosity assumption in the early 1960s. Efforts were directed towards numerical analyses of more realistic flow situations including shear-thinning and non-isothermal effects. The complexity and accuracy of the analysis increase when the non-Newtonian flow behavior of polymer melts is included. Pseudoplastic behavior complicates the mathematical model such that the governing flow equations must be solved numerically, and exact closed-form analytical solutions are no longer possible. The viscosity being dependent on the shear rate, the drag and pressure flows are coupled. For multidimensional flows, complexity is increased further by the combined effect of shear in the down- and cross-channel directions, which couples the flow components in these directions.

### 4.1. One-Dimensional Non-Newtonian Down-Channel Flows

Initially, numerical solutions were obtained for the isothermal down-channel flow of a shear-thinning fluid between parallel plates [85,86,87,88,89,90,91,92,93,94,95,96]. Even for a one-dimensional fully developed temperature-independent laminar flow of an incompressible power-law fluid between two parallel plates, no exact closed-form analytical solution has been found to date [7]. Details on this type of flow are provided in Table 2 (1D_d).

Several studies have presented analytical solutions for the combined drag and pressure flow of power-law fluids based on a variety of mathematical approaches. All of these require the integration constants to be evaluated numerically. Examples include approaches by Rotem and Shinnar [86], Clyde and Holmes-Walker [87], Weeks and Allen [88], Krüger [89], Kroesser and Middleman [90], Flumerfelt et al. [91], and Tadmor and Gogos [4]. A closed-form solution with a reduced accuracy at small down-channel pressure gradients was derived by Rauwendaal [7]. Recently, Steller and Igwo [92] proposed approximate equations for the integration constants.

The solution to the flow equation depends on the sign and the magnitude of the pressure gradient, which affect the shape of the velocity and, thus, the shear-rate profile. In total, results were presented for four types of flow condition, including pressure-generating and pressure-consuming flows. Further distinctions were made based on the sign of the shear-rate profile.

**Figure 7:**
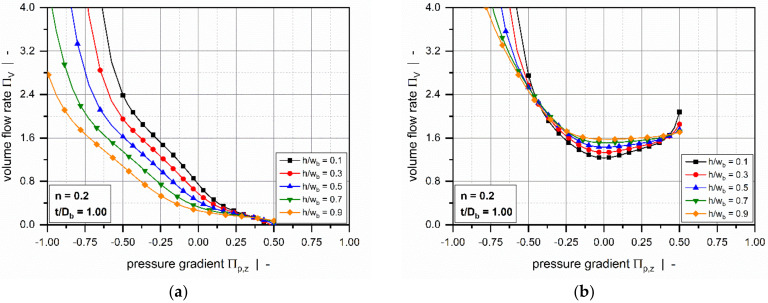
Influence of the power-law index on the fully developed one-dimensional down-channel flow of a power-law fluid under isothermal conditions. The dimensionless volume flow rate $\Pi_{V}$ as a function of the dimensionless pressure gradient $\Pi_{p,z}$ (**a**), and the dimensionless dissipation $\Pi_{Q}$ as a function of the dimensionless pressure gradient $\Pi_{p,z}$ (**b**).

Using the finite-difference method, Roland and Miethlinger [93] solved a dimensionless form of the model in Table 2 (1D_d), which was shown to be governed by two physically independent dimensionless input parameters: (1) the power-law index n, and (2) a dimensionless down-channel pressure gradient $\Pi_{p,z}$; Figure 7 illustrates their effects on the dimensionless flow rate $\Pi_{V}$ and the dissipation $\Pi_{Q}$.

For Newtonian fluids, the widely known linear relationship between flow rate and pressure gradient is evident: for $\Pi_{p,z\;}=0$ (drag flow), the curve satisfies $\Pi_{V\;}=1$, while for $\Pi_{p,z\;}=1$, the zero-throughput condition is fulfilled. The curve becomes increasingly nonlinear and pressure-sensitive with decreasing power-law index. For a positive dimensionless pressure gradient, the flow rate decreases with decreasing power-law index. The opposite behavior is observed when the dimensionless pressure gradients become negative. A minimum in dimensionless dissipation is given for pure drag flow conditions. In general, the dimensionless dissipation increases if the pressure flow contributes to the flow characteristics; that is, the higher the dimensionless pressure gradient, the more pronounced the frictional heat generation.

For Newtonian fluids, the discharge rate results from linear superposition of a drag and a pressure flow. Such a treatment is invalid for shear-thinning fluids, where the flow components are interrelated and the fluid velocities are more complex than the drag and pressure velocity profiles superimposed. Jacobi [10] applied the superposition principle for shear-thinning fluids by introducing a power-law model in the pressure flow term and adding it to the drag flow. The validity of this simplified approach was examined by Kroesser and Middleman [90], who compared the relative errors between numerical solutions of the combined drag and pressure flow and those resulting from the superposition principle. It was shown that, depending on the pressure characteristics of the melt-conveying zone, linear superposition of the flows may cause substantial errors in the prediction of the throughput.

All of the abovementioned numerical studies omitted the influence of the screw flights on the down-channel flow. When wall effects are taken into account, the mathematical problem, described by the equations and boundary conditions in Table 2 (1D_e), involves a nonlinear partial differential equation. Wheeler and Wissler [97] and Palit and Fenner [98] presented numerical solutions, using the finite difference and finite element methods, respectively. Middleman [99] independently solved the drag and pressure flows of a power-law fluid in a rectangular flow channel, and calculated shape factors for the drag and pressure flows; for both flow components, he demonstrated that the rate-limiting influence of the walls, as described by Equations (47) and (48) for Newtonian fluids, increases the more shear-thinning the polymer melt. This effect was particularly pronounced for the pure pressure flow.

When temperature effects are included, the complexity of the mathematical problem increases further. Since velocity and temperature fields are coupled, the interconnected influence of shear rate and temperature on viscosity must be considered. Colwell and Nicholls [100] investigated a temperature-dependent flow of a non-Newtonian fluid between two parallel plates maintained at arbitrary temperatures. Taking viscous heat generation and conduction into account, numerical solutions were presented for the temperature and velocity profiles over the channel depth. For power-law fluids, the model is defined by the equations and boundary conditions in Table 2 (1D_f).

### 4.2. Two-Dimensional Non-Newtonian Flows in Screw Channels of Infinite Width

The shear-thinning behavior of polymer melts couples the down- and cross-channel flows via the viscosity function. The ratio between down- and cross-channel flows is governed by the screw–pitch ratio $t/D_{b}$. The greater the helix angle, the more pronounced the transverse flow. After one-dimensional flows had been mostly worked out, efforts were directed towards analysis of two-dimensional flows of shear-thinning fluids in infinitely wide screw channels (Figure 4b). Solutions were obtained for both thermally fully developed and developing flows, whose equations and boundary conditions are summarized in Table 3.

#### 4.2.1. Fully Developed Flows

Models of a hydrodynamically and thermally fully developed two-dimensional flow of a power-law fluid under both isothermal and non-isothermal conditions have appeared in various articles [93,101,102,103,104,105,106] and textbooks [3,7,14]; these types of flow are governed by the models in Table 3 (2D_b and 2D_c, respectively).

The first major contribution that included non-isothermal, shear-thinning effects was presented by Griffith [101], who ignored the effects of down-channel heat convection and conduction, and assumed the temperature distribution along a streamline to be constant. Furthermore, the temperatures of the barrel and screw surfaces were considered to be equal. Griffith thus intended to counterbalance the absence of thermal cross-channel convection, which takes place in real three-dimensional recirculating flows.

Similarly, Zamodits and Pearson [102] calculated screw characteristics for various screw geometries and rheological parameters under both isothermal and non-isothermal conditions; rather than following Griffith’s approach, they used an adiabatic temperature boundary condition at the screw. Later, Steller [103,104] developed an alternative method for predicting the flow of power-law and Ellis fluids in infinitely wide screw channels. His algebraic expressions for the velocity profiles require numerical evaluation of the integration constants, which were approximated recently by Steller and Igwo [92].

**Figure 8:**
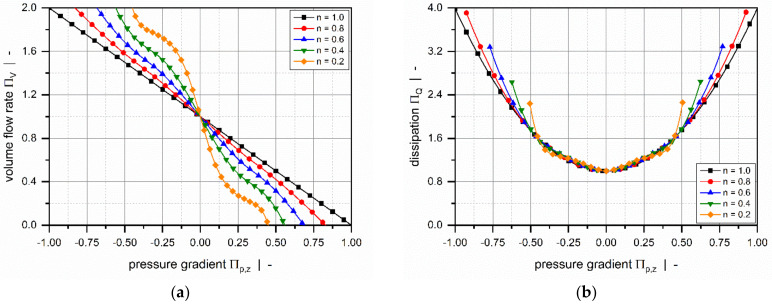
Influence of the power-law index on a fully developed two-dimensional flow of a power-law fluid in a screw channel of infinite width under isothermal conditions. The dimensionless volume flow rate $\Pi_{V}$ as a function of the dimensionless pressure gradient $\Pi_{p,z}$ (**a**), and the dimensionless dissipation $\Pi_{Q}$ as a function of the dimensionless pressure gradient $\Pi_{p,z}$ (**b**).

**Figure 9:**
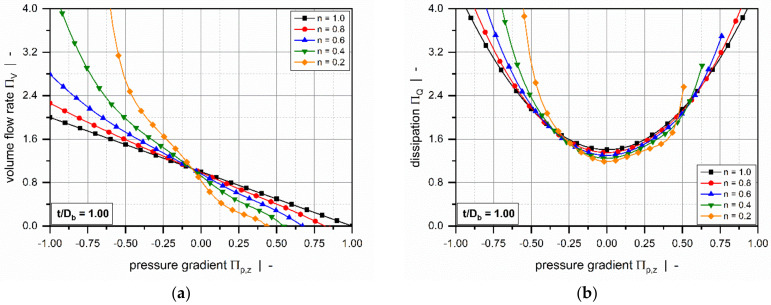
Influence of the power-law index on a fully developed two-dimensional flow of a power-law fluid in a screw channel of infinite width under isothermal conditions. The dimensionless volume flow rate $\Pi_{V}$ as a function of the dimensionless pressure gradient $\Pi_{p,z}$ (**a**), and the dimensionless dissipation $\Pi_{Q}$ as a function of the dimensionless pressure gradient $\Pi_{p,z}$ (**b**).

Roland and Miethlinger [105] investigated the usefulness of selected numerical techniques in solving the nonlinear boundary value problem defined in Table 3 (2D_b). Analogously to previous approaches [101,102], they solved a dimensionless form of the problem, which was shown to depend on three physically independent dimensionless input parameters [93,106]: (1) the screw–pitch ratio $t/D_{b}$, (2) the power-law index n, and (3) a dimensionless down-channel pressure gradient $\Pi_{p,z}$. Figure 8 and Figure 9 illustrate the influences of these parameters on the dimensionless flow rate $\Pi_{V}$ and the dissipation $\Pi_{Q}$.

Comparing the results to the numerical solutions in Figure 7 reveals of the following differences: (1) The drag flow at $\Pi_{p,z\;}=0$ decreases with decreasing power-law index and increasing screw–pitch ratio. The latter is a measure of the influence of the transverse flow. Note that if transverse flow is ignored ($t/D_{b}=0$), the curves satisfy $\Pi_{V\;}=1$ at $\Pi_{p,z\;}=0$. (2) For positive and slightly negative dimensionless pressure gradients, the flow rate decreases with increasing screw–pitch ratio; this behavior changes if a critical negative pressure gradient is exceeded. (3) Viscous dissipation increases with increasing helix angle of the screw.

#### 4.2.2. Developing Flows

When the polymer melt is subjected to strong dissipation and conductive heating, thermal convection in the down-channel direction becomes significant, and must be considered. Due to the temperature sensitivity of the viscosity, the local temperature change additionally affects the velocity field, which was traditionally assumed to adjust instantaneously to the local temperature field. Two-dimensional thermally developing flows of power-law fluids in infinitely wide screw channels have been solved by many researchers, including Yates [56], Fenner [107], Agur and Vlachopolous [108], Bruker et al. [109], and others [110,111,112]. Details on the governing equations and boundary conditions are shown in Table 3 (2D_d).

For a wide range of operating conditions, Equation (34) is parabolic in the z-direction, which allows use of a marching technique to obtain the solution for the entire flow domain. The procedure requires definition of an initial condition for the temperature at the inlet of the screw channel, while no boundary conditions must be specified at the outlet. This technique fails to simulate the fluid flow when the down-channel velocity becomes negative, as is the case for large back pressures or low throughputs. Solutions to these numerical problems were presented by Elbirli and Lindt [113], and by Chiruvella et al. [114]. The former coupled the heat transfer and residence time characteristics of thermally developing extruder flows to calculate stable solutions even under appreciable pressure backflow; the latter proposed two solution methods dealing with the same problem. One scheme was based on including the down-channel thermal diffusion, making the problem elliptic, while the other scheme used a different coordinate system.

### 4.3. Three-Dimensional Non-Newtonian Flows in Screw Channels of Finite Width

The infinite-channel-width assumption provides a reasonable approximation of the screw geometry for shallow screw channels with small $h/w_{b}$. The transport processes in extruders, however, are three-dimensional. In a screw channel of finite width (Figure 4a), the screw flights generate a recirculating cross-channel flow. A major shortcoming of the previously mentioned two-dimensional models is that the effect of the screw flights is included by mass conservation considerations only, and the recirculating transverse flow with two non-vanishing velocity components $v_{x}$ and $v_{y}$ is not captured. Especially for deep metering channels, the screw flights may affect the flow pattern and heat transfer significantly. To date, only a few studies have computed three-dimensional velocity and temperature fields for both thermally fully developed and developing flows. An overview of mathematical models of a three-dimensional flow of a power-law fluid is given in Table 4.

#### 4.3.1. Fully Developed Flows

One of the first analyses of a hydrodynamically and thermally fully developed flow of a power-law fluid in a metering channel of finite width was published by Martin [115]; using finite-difference techniques, he solved the model in Table 4 (3D_b) for various channel aspect ratios.

**Figure 10:**
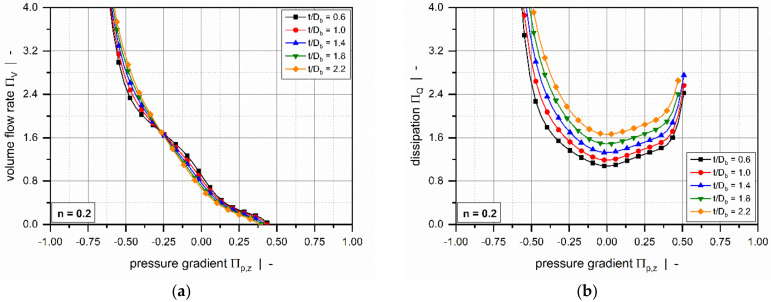
Influence of the power-law index on a fully developed three-dimensional flow of a power-law fluid in a screw channel of finite width under isothermal conditions. The dimensionless volume flow rate $\Pi_{V}$ as a function of the dimensionless pressure gradient $\Pi_{p,z}$ (**a**), and the dimensionless dissipation $\Pi_{Q}$ as a function of the dimensionless pressure gradient $\Pi_{p,z}$ (**b**).

**Figure 11:**
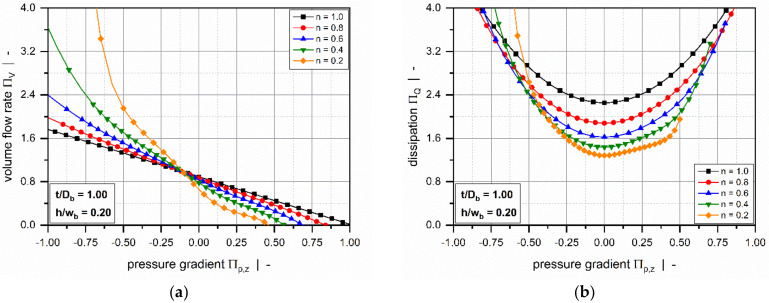
Influence of the aspect ratio on a fully developed three-dimensional flow of a power-law fluid in a screw channel of finite width under isothermal conditions. The dimensionless volume flow rate $\Pi_{V}$ as a function of the dimensionless pressure gradient $\Pi_{p,z}$ (**a**), and the dimensionless dissipation $\Pi_{Q}$ as a function of the dimensionless pressure gradient $\Pi_{p,z}$ (**b**).

Still retaining the isothermal assumption, Marschik et al. [116,117] recently transformed the equations and boundary conditions in Table 4 (3D_a) into a dimensionless form, showing that the model is governed by four physically independent dimensionless input parameters: (1) the aspect ratio $h/w_{b}$, (2) the screw-pitch ratio $t/D_{b}$, (3) the power-law index n, and (4) a dimensionless down-channel pressure gradient $\Pi_{p,z}$. The model was then solved using the finite volume method to evaluate the dimensionless volume flow rate $\Pi_{V}$ and the dissipation $\Pi_{Q}$ for various operating conditions, as demonstrated in Figure 10 and Figure 11.

The influence of the flight flanks is dominated by the aspect ratio of the screw channel $h/w_{b}$. For most pressure-generating and pressure-consuming flows, the flow rate decreases with increasing aspect ratio. The rate-limiting effect is particularly pronounced in overridden zones. In most standard extruder screws, the channel aspect ratio ranges from $0.05\leq h/w_{b}\;\leq 0.15,$ and the restricting effect of the flights on the flow rate is limited. However, for more advanced extruder screws, such as barrier and wave-dispersion screws, the ratio of channel depth to channel width may significantly exceed $h/w_{b}\;\geq 0.15$; in this case, the screw flights substantially affect the velocity and temperature fields. Dimensionless dissipation increases with increasing aspect ratio. The reason for this is that close to the walls high shear and dissipation rates prevail, which becomes increasingly important for narrower channels. Especially for large negative pressure gradients, this effect is inverted due to pronounced changes in flow rate.

#### 4.3.2. Developing Flows

Three-dimensional thermally developing flows of power-law fluids in screw channels of finite width are governed by the equations and boundary conditions shown in Table 4 (3D_c). For a long time, solutions to this problem remained elusive due to numerical instabilities caused by convection terms in the energy equation. With the advent of more sophisticated numerical methods, a number of studies have been able to compute the three-dimensional fluid flow and heat transfer with various boundary conditions.

Accounting for thermal convection in the down- and cross-channel directions, Syrjälä [57,118,119] demonstrated the influence of the recirculating transverse flow on the temperature distribution in the screw channel. The recirculatory motion of the polymer melt conveys fluid particles from the region near the heated barrel down the channel and back again. In combination with viscous heating effects, this mechanism reduces the temperature variation over the channel depth compared to a two-dimensional flow in an infinitely wide channel. Further numerical results were presented by Lawal and Kalyon [120], Sastrohartono et al. [121], and Ghoreishy et al. [122,123].

All of these theories used a constant barrel temperature and considered either isothermal screw surfaces or adiabatic boundary conditions. In all cases, the solution procedure for the parabolized equation system was based on a finite element scheme in combination with marching in the down-channel direction. The three-dimensional problem was thereby reduced to a series of two-dimensional problems solved stepwise in each cross-channel plane. A variety of methods were used to eliminate the numerical instabilities arising from the dominant convection terms, such as the standard Galerkin finite element method and a streamline upwind Petrov–Galerkin formulation.

## 5. Approximate Methods

In spite of their high relevance, the usefulness of numerical methods for practical screw design is limited, as they are complex, often require high computational effort, and tend to be time-consuming. Although recent progress in computer technology has pushed back computational barriers to solving equation systems with various degrees of nonlinearity, solving the complex flow conditions in single-screw extruders remains time-consuming, especially when more realistic models are considered.

To remove the need for numerical techniques, several studies have proposed approximate methods for predicting the flow in metering channels by either (1) introducing correction factors to the Newtonian pumping model described in Equation (46), or (2) developing new analytical regressions for various target variables. These methods typically allow for faster analysis of the conveying behavior, and are therefore particularly useful in design and optimization studies, in which multiple modeling setups are compared. For these applications, the accuracy of approximate equations is often sufficient. In addition, the use of cost-intensive simulation software can be avoided.

Most of these approaches were designed to approximate the numerical solutions for a hydrodynamically and thermally fully developed flow of a power-law fluid under isothermal conditions. Approximations were developed for a one-dimensional down-channel flow (Table 2, 1D_d), a two-dimensional flow in an infinitely wide channel (Table 3, 2D_b), and a three-dimensional flow in a channel of finite width (Table 4, 3D_a). Figure 7, Figure 8, Figure 9, Figure 10 and Figure 11 illustrate numerical results for these types of flow, while Table 5 presents an overview of existing approximate methods. For detailed information on the flow conditions considered in each case, see Section 4.

Pioneering theories were published by Krüger [89] and Booy [124], who presented a method for selecting an effective viscosity for the pressure flow term in the Newtonian pumping model. While the former used numerical results obtained for the one-dimensional down-channel flow of a power-law fluid under isothermal conditions, the latter extended the analysis to two-dimensional flows in infinitely wide screw channels. Effective viscosity is the Newtonian viscosity that yields the same performance as if power-law fluids were used. A refined approach was presented by Rauwendaal [7,125], who derived correction factors for the drag and pressure flows, which can be applied to pressure-generating metering zones with helix angles of $15{}^{\circ}\leq \varphi_{b}\leq 25{}^{\circ}$. Similarly, Rauwendaal approximated the numerical results for a two-dimensional flow.

A similar method was presented by Spalding and Campbell [6,126], who proposed a correction factor for the drag flow based on their numerical results for a three-dimensional flow. Since the pressure flow term remained unmodified, this approach leaves some matters unaddressed. Kim and Kwon [127] suggested a different approach to determining screw characteristic curves for three-dimensional flows with the aid of a total shape factor defined by the ratio of numerically determined three- and two-dimensional flow rates. Although the work provides numerical solutions for the shape factor, an analytical approximation is only available for a Newtonian fluid.

Another method was proposed by Potente [128,129]; using numerical solutions obtained for various flow situations, he derived analytical regressions to estimate the flow rate and power consumption of the metering zone. Initially, approximations were developed for the one-dimensional down-channel flow of a power-law fluid under isothermal conditions [128]. Later, the regressions were improved to predict the flow in a screw channel of infinite width [129]. While the throughput approximation is valid for dimensionless flow rates of $0.55\leq \Pi_{V}\leq 1.45$ and pitch angles of $0{}^{\circ}\leq \varphi_{b}\leq 17.65{}^{\circ}$, the power consumption approximation holds for dimensionless flow rates of $0.55\leq \Pi_{V}\leq 1.25$.

Potente’s regressions were further refined in two PhD theses: Effen [130] extended the application range of the throughput model by considering dimensionless flow rates of $0.1\leq \Pi_{V}\leq 2.0$, power-law indices of 0.2≤n≤1.0, and screw–pitch ratios of $0.8\leq t/D_{b}\leq 2.0$. A drawback of this approach is that the regression coefficients depend on the input parameter values, causing the model to exhibit undefined and discontinuous regions. Obermann [131,132] developed a more accurate model of the power consumption by approximating numerical results for a three-dimensional flow.

To provide melt-conveying models that are continuous across their full application range, we have recently proposed a hybrid modeling approach [133]. The novelty of our method lies in the construction of analytical approximation equations from a large number of numerical solutions to scaled flow equations by using symbolic regression based on genetic programming. Unlike other regression techniques, such as linear or polynomial regression, this modeling approach requires neither the model’s structure nor its parameters to be predefined. Rather, the approach applies evolutionary computation methods to uncover mathematical relationships based on comprehensive datasets. The usefulness of this modeling approach was demonstrated for various polymer-processing problems [134,135].

Roland [136] proposed approximate equations for flow rate and viscous dissipation, taking a one-dimensional flow of power-law fluid into account. Pachner et al. [137] and Roland and Miethlinger [93] each presented analytical regressions for a two-dimensional flow. The throughput model is valid for 0.2≤n≤0.9, $0.75\leq t/D_{b}\leq 2.0$, and $0\leq \Pi_{V}\leq 2.0$, while the dissipation model holds for 0.2≤n≤1.0, $0.5\leq t/D_{b}\leq 2.0$, and $0\leq \Pi_{V}\leq 2.0$. Roland et al. [106] further optimized the equations to additionally include screw sections subjected to large negative dimensionless pressure gradients, as typically found in strongly overridden wave- or energy-transfer screws. These optimized models for the flow rate and dissipation are valid for 0.2≤n≤1.0, $0.6\leq t/D_{b}\leq 2.4$, and $1.0\leq \Pi_{p,z}\leq \mathrm{variable}$. The upper boundary for the dimensionless down-channel pressure gradient was adjusted case by case to exclude negative volume flow rates $\left(\Pi_{V,min}\geq 0\right)$.

Using the same methodology, Marschik et al. [116,138,139] and Roland et al. [117] approximated the numerical solutions for the flow rate and viscous dissipation of a three-dimensional flow of a power-law fluid. The approximate equations take into account the shear-thinning flow behavior of the polymer melt, the influence of transverse flow, and the effect of the screw flights. The regression models are valid for 0.2≤n≤1.0, $0.5\leq t/D_{b}\leq 2.0$, and $0.05\leq h/w_{b}\leq 0.5$. Similar to the previous approach, the range of the dimensionless down-channel pressure gradients was adjusted as a function of the power-law index. The validity of the two- and three-dimensional melt-conveying models was tested against experimental extrusion data measured for both standard and high-performance screws [140,141,142]. Recently, the usefulness of the symbolic regression analysis was analyzed in a comparative study that investigated various data-based modeling approaches [143].

While all of the aforementioned approaches analyzed polymer melt flows under isothermal conditions, a few studies [144,145,146,147] have proposed a lumped-parameter method for approximating the axial temperature along the extruder screw. Rather than resolving spatial parameter variations over the cross-section, this method divides the screw channel into short axial segments, in which a lumped form of the energy equation based on a mean melt temperature is solved.

To demonstrate the validity of the selected approximation methods, we compared their accuracy in predicting numerical solutions for the flow rate $\Pi_{V}$ obtained for a three-dimensional flow of a power-law fluid in a screw channel of finite width, as mathematically described in Table 4 (3D_a). Table 6 provides an overview of the three-, two-, and one-dimensional approaches investigated in this study. Ignoring the effect of the shear-thinning flow behavior, the sixth approach is the Newtonian pumping model given in Equation (46). Except for the first method, which inherently considers the effect of the flight flanks, all remaining models were additionally corrected by using the shape factors in Equations (47) and (48). This step required the nonlinear regressions in the fourth and fifth approaches to be linearized.

The prediction accuracy of the approximations in Table 6 was evaluated by using the dataset generated in the construction of our throughput model [116]. Considering the different scopes of the methods, this dataset was subdivided into three subsets according to the validity ranges of the models. Each of these consisted of numerous physically independent design points defined by n, $t/D_{b}$, $h/w_{b}$, $\Pi_{p,z}$, and the corresponding numerical solution for $\Pi_{V}$. For each setup, we additionally applied the approximations in Table 6 to estimate the flow rate.

Table 7 illustrates the parameter ranges of the datasets and the approximate methods applied in each case. For all datasets, the upper boundary for $\Pi_{p,z}$ was adjusted case by case to avoid negative volume flow rates $\left(\Pi_{V,min}\geq 0\right)$. The first dataset is based on the scope of the three-dimensional approach (Model 1), and includes 77,411 design points. The second and third datasets were obtained by restricting the ranges of $t/D_{b}$ and $\Pi_{V}$ according to the validity ranges of Effen’s and Rauwendaal’s methods (Model 3 and Model 2), yielding 57,025 and 26,571 design points, respectively.

Table 8 illustrates the accuracy of the approximate methods by comparing (1) mean absolute errors between the numerical and approximated results (MAE), and (2) coefficients of determination ($R^{2}$). Including the shear-thinning flow behavior, the effect of transverse flow, and the influence of the flight flanks, Model 1 shows the highest accuracy in the prediction of the numerical solutions. Significant differences in the quality measures are observed in the case of the two-dimensional methods. While Model 4 performs well for all datasets, Model 3 exhibits pronounced errors in the approximation of the three-dimensional flow. Differences between Model 1 and Models 2–4 are caused by the specific way in which the influence of the screw flights is considered. While the two-dimensional methods apply the shape factors originally developed for Newtonian fluids, Model 1 inherently includes the influence of the shear-thinning flow behavior on the wall effects. Prediction accuracy is further decreased if the effect of transverse flow is ignored (Model 5) or Newtonian flow behavior is assumed (Model 6).

## 6. Leakage Flow

The effect of the flight clearance on fluid flow and heat transfer in the metering zone has been investigated since the earliest studies of screw pumps. For conventional extruder screws, a typical value of the flight clearance is 0.1% of the barrel diameter ($\delta =0.001\;D_{b}$). This rule is invalid in the case of high-performance screws, as their screw flights are often strategically undercut in order to promote transverse mixing. In this case, leakage flow was shown to play a significant role in the analysis of melt-conveying zones.

The first Newtonian theory to account for the effect of the flight clearance was published anonymously [68], and later revisited by Rowell and Finlayson [69], Carley and Strub [19], and Carley et al. [71]; using the same approach, they approximated the annular clearance between flight land and barrel surface using two parallel plates, and assumed the leakage flow to be a pressure flow through an infinitely wide slit. The rate of leakage flow was then subtracted from the net rate of discharge obtained from the traditional Newtonian pumping model in Equation (46).

Gore and McKelvey [83] included the effect of the flight clearance on the drag flow by using an effective channel depth in the integration of the down-channel velocity. At the same time, Mohr and Mallouk [75] refined the existing theories by additionally considering the drag-induced cross-channel pressure gradient. A complete description of the Newtonian analysis was given by Tadmor and Klein [1]. Ignoring the rate-limiting influence of the screw flights, the total output can be obtained from Equations (56) and (57):

(56) $$\dot{V}=\frac{i\;w_{b}\;h\;v_{b,z}}{2}\left(1-\frac{\delta }{h}\right)-\frac{i\;w_{b}\;h^{3}\;}{12\;\eta }\frac{dp}{dz}\left(1+f_{L}\right)$$

(57) $$f_{L}={\left(\frac{\delta }{h}\right)}^{3}\frac{e}{w_{b}}\frac{\eta }{\eta_{f}}+\frac{\left(1+\frac{e}{w_{b}}\right)\left[\frac{6\;\eta \;v_{b,z}\left(h-\delta \right)}{h^{3}\;dp/dz}+\frac{1+e/w_{b}}{tan^{2}\left(\varphi_{b}\right)}\right]}{1+\frac{\eta_{f}}{\eta }{\left(\frac{h}{\delta }\right)}^{3}\frac{e}{w_{b}}}$$

Including the shear-thinning flow behavior of polymer melts, Rauwendaal et al. [148,149] presented a numerical analysis of leakage flow based on the finite difference method. To analyze the effect of the flight clearance on the overall flow behavior, they coupled the flows in the metering channel and in the flight gap by correcting the condition for the net cross-channel flow in (42). Assuming a fully developed isothermal flow of a power-law fluid, both problems were treated as a two-dimensional flow in an infinitely wide channel. The effect of the leakage flow on the throughput was large for highly shear-thinning polymer melts and pronounced flight clearances ($\delta =0.004\;D_{b}$). For a power-law fluid with n=0.3, the drag flow rate and the maximum pressure-generating capability decreased by roughly 7% and 20%, respectively, when the flight clearance was increased from $\delta =0.001\;D_{b}$ to $\delta =0.004\;D_{b}$. Note that the former represents a typical value of the flight clearance in standard screws, while the latter can be found in more advanced screw designs. For a Newtonian fluid, in contrast, the throughput was lowered by ~10% at medium values of the pressure gradient for the same settings. The velocity profiles in the flight gap were almost linear for a Newtonian fluid, while the contribution of the pressure gradients to the leakage flow was significant for fluids with small power-law indices. Furthermore, the total power consumption of the screw was shown to be affected considerably by the power consumption in the flight clearance.

Taking non-isothermal effects into account, Meyer et al. [150] investigated the temperature development in the flight clearance. Assuming a drag flow between two parallel plates maintained at isothermal temperatures, they showed that for both Newtonian and power-law fluids the thermal development length is generally smaller than the available gap length. In the prediction of the velocity and temperature profiles at the exit of the clearance, convective heat transport can therefore be ignored, and the flow can be treated as thermally fully developed.

To examine the influence of leakage flow on the temperature distribution in the screw channel, Pittman and Rashid [151] numerically studied the heat transfer in the two-dimensional recirculation flow over the channel cross-section of a twin-screw extruder. In their simplified approach, the influence of the down-channel velocity component was omitted. Alongside viscous heat generation and heat transport by conduction and convection, the authors included the sensitivity of the viscosity to shear rate and temperature using a temperature-dependent power-law model. To specify the flow conditions in the leakage gap, a drag flow velocity profile was considered. The governing flow equations were solved using the finite element method.

Rauwendaal [152] expanded on the two-dimensional theory by including the previously ignored down-channel velocity component; similarly, he investigated the influence of screw geometry, materials, and processing conditions on the velocity, pressure, and temperature fields in the screw channel using the finite element method. The convective term in the energy equation was stabilized by a streamline upwind Petrov–Galerkin formulation. The results showed that the melt temperatures are lower in the flight clearance than in the screw channel, since the high level of viscous heat generated in the clearance is conducted away to the barrel due to its close proximity. In addition, the maximum temperature and the area of the high-temperature region in the channel were shown to increase with larger flight clearance.

Recently, we solved a local formulation of the two-dimensional isothermal flow of a power-law fluid through the flight clearance [153]; using the shooting method, our analysis evaluated flow rate and dissipation in the leakage gap for a variety of screw designs and processing conditions.

## 7. Curved Channel Systems

For shallow screw channels with low $h/D_{b}$, the error introduced by the flat-plate approximation is small (Figure 4). The influence of the channel curvature can therefore be ignored without a significant loss in prediction quality. Closer attention must be paid to deep melt channels with pronounced $h/D_{b}$. In this case, the predicted conveying behavior might be affected substantially by the choice of reference system. A number of studies analyzed the influence of channel curvature on the flow of both Newtonian and power-law fluids [37,43,44,154]. A comparison of various channel configurations showed that while the error of the flat-plate approximation amounts to up to 10% for shallow screw sections ($h/D_{b}\approx 0.05$), the flattened channel representation can give rise to errors greater than 30% for deep channels ($h/D_{b}\approx 0.30$), depending on the shear-thinning nature of the polymer melt and the magnitude of the pressure gradient.

To account for the effect of channel curvature, approaches were introduced for deep channels that are based either on cylindrical polar or helical coordinates. One of the first theoretical approaches that avoided the flat-plate model was presented by Squires [20], who derived a correction factor for the drag flow in the traditional Newtonian pumping model (Equation (46)), which is valid for the limiting case of zero helix angle and infinite aspect ratio. Booy [155] extended the theory by including the effect of helix angle and its variations with the radius; his model consisted of a screw channel bounded by the cylindrical barrel and screw root surfaces, as well as by the two sides of a helical flight. Assuming a fully developed flow in an infinitely wide channel, Booy solved the governing flow equations with two non-zero-velocity components in the tangential and axial directions, defined in cylindrical polar coordinates. To consider the effect of both channel curvature and helix angle, correction factors for the drag and pressure flows in the traditional melt-conveying model were computed. Tadmor and Klein [1] additionally provided drag and pressure shape factors for the tangential flow of Newtonian fluids.

While most of the aforementioned analyses were based on the constant-viscosity assumption, later studies included the shear-thinning flow behavior of polymer melts based on various mathematical approaches [156,157,158,159,160,161]. Using the finite difference method, Dyer [156] investigated a three-dimensional non-isothermal flow of a temperature-dependent power-law fluid in a curved screw channel in the absence of leakage flow. Steller [157] presented models for a fully developed flow of an Ellis fluid in a curved screw channel under both isothermal and non-isothermal conditions. Since the channel was assumed to be infinitely wide, the flow was considered to be two-dimensional, with two non-zero-velocity components in the tangential and axial directions. In this non-isothermal theory, viscous dissipation, heat conduction in the radial direction, and heat convection in the tangential direction were included. Both of these studies [156,157] defined the governing flow equations in cylindrical polar coordinates.

Lim et al. [158] proposed a partial periodic unit technique in combination with the finite element method to reduce the computational time for a three-dimensional flow of a power-law fluid in a helical screw channel. Spalding et al. [159] carried out three-dimensional finite element simulations for a helical metering section, considering combined drag and pressure flow. Conzen [160] performed similar investigations using the finite volume method.

Several studies have used a helical coordinate system to analyze the flow of polymer melts in deep, curved screw channels [27,28,29,30,31,32,33,34,35,36]. Equivalent definitions of a non-orthogonal helical coordinate system were presented by Zamodits [27] and Tung and Lawrence [28]; while the former was applied to model curved extruder screws, the latter was used for static mixers. Nebrensky et al. [29] set up a variational formulation of a developed flow and heat transfer in a single-screw extruder as a two-dimensional problem. A function applicable to non-isothermal flows of generalized Newtonian fluids was expressed in a non-orthogonal helical coordinate system. Hami and Pittman [30] solved the variational problem for an isothermal Newtonian flow for both shallow and deep channels using the finite element method. Choo et al. [31] extended the analysis by providing finite element predictions for an isothermal flow of power-law fluids. Wang and Andrews [32] formulated the equations of continuity and motion in an alternative helical system, and used the finite difference method for solving fully developed flows of Newtonian fluids. Blyth and Pozrikidis [33] performed a perturbation analysis in non-orthogonal helical coordinates to independently analyze the drag and pressure flows in curved screw channels.

Introducing further physical simplifications, Sanjabi et al. [34,35] mathematically described the three-dimensional helical flow of a temperature-dependent power-law fluid in extruders. The governing flow equations, which additionally account for tapered screw sections, were first developed in cylindrical polar coordinates, and then transformed into a helical system. Solutions were computed with the aid of an iterative computational algorithm based on the shooting method. Combing helical coordinates with viscoelastic flow behavior, Vachagina et al. [36] simulated a non-isothermal flow of a Giesekus fluid flow in the melt-conveying zone of a single-screw extruder.

## 8. Conclusions

We have reviewed the developments in the modeling and simulation of the melt-conveying zones of single-screw extruders from a methodological perspective. The melt-conveying process is a critical processing step in a variety of extrusion operations, and has therefore received significant attention in the literature. Since the 1920s, the complexity and accuracy of melt-conveying models has increased significantly, as the constraints of traditional modeling assumptions and simplifications have been increasingly relaxed. This progress has come in tandem with developments in computer power and more sophisticated modeling techniques. Assuming a fully developed steady-state laminar flow of an incompressible fluid, the first analyses provided closed-form analytical solutions for the conveying behavior of Newtonian fluids with temperature-independent viscosity. Using numerical methods, latter step-by-step studies included the effect of the shear-thinning and non-isothermal behavior in one-, two-, and three-dimensional flows in channels of both infinite and finite widths.

Numerous flow situations based on various geometric and physical conditions have been analyzed. While the solution methods—including analytical, numerical, and approximate approaches—have changed over the years, the mathematical models are still set up using the conservation equations of mass, momentum, and energy in combination with constitutive equations and boundary conditions. Historically, most theories treated the polymer melt as a purely viscous shear-thinning fluid, and approximated the helical screw channel using the flat-plate model. While these assumptions yield reasonable results for a variety of processing conditions, they fail to deliver accurate solutions when more complex materials (e.g., filled polymers or polymer suspensions) and screw geometries (e.g., barrier or wave-dispersion screws) are considered. A thorough understanding of these flow situations would require computational analysis of helical extruder channel flows based on advanced material models that include viscoelastic flow behavior and wall-slippage effects. While some of these problems have already been discussed for selected boundary conditions in the literature [50,51,52,58,59,60,61,62,63,64,65,66], their analysis often remains restricted by the computational power available.

From a methodological viewpoint, the existing melt-conveying models can be classified into exact analytical, numerical, and approximate approaches. Depending on the problem at hand, all of these provide specific advantages and disadvantages. Analytical solutions are usually stable and fast to compute. Since parameter dependencies are often expressed explicitly, they provide a clear view of how input parameters affect target variables; as a result, they are particularly useful in design and optimization studies. While exact analytical solutions were only obtained for Newtonian fluids, approximate solutions were constructed for all types of temperature-independent shear-thinning flows. The latter were often presented in the form of correction factors or regression models, which can also be combined to simultaneously consider various effects within one approach (e.g., the influence of flight flanks and leakage flow). Numerical results, in contrast, are given by discrete values rather than complete mathematical expressions. A major advantage of numerical methods is their capability of handling large equation systems with different degrees of nonlinearity; in contrast to their counterparts, they allow a more accurate assessment of the flow phenomena in the screw channel.

Although most melt-conveying models were originally designed for single-screw extruders, some of them may also be applied to injection molding machines. In this case, the velocity boundary conditions must be rewritten to include the axial retraction speed of the screw, as suggested in [117].

Despite the vast amount of literature, more research must be carried out in order to achieve the goal of accurately predicting the conveying characteristics of single-screw extruders. Further sophisticated numerical computations of three-dimensional flows with more realistic conditions are required in order to increase our understanding of the transport processes. These especially include non-isothermal developing flows and partially filled systems.

A drawback of most shear-thinning theories is the power law used in the modeling of the viscosity behavior. Since this approach works well only within a certain range of shear rates, more sophisticated multiparameter models must be used to accurately predict the viscosity behavior of the polymer melt in both the Newtonian and the shear-thinning regimes. Even in conventional screw designs, the flow field is subject to shear rates ranging over several orders of magnitude, taking the diverse channel depths in the screw channel and in the flight clearance into account. These variations are typically not captured by the power law. To remove its limitations, numerous viscosity functions of increased complexity are available in the literature [4]. Examples include the Carreau–Yasuda model, the Cross model, or the Ellis model. For most conditions, these models provide a more accurate representation of the viscous flow behavior.

A fundamental shortcoming of the traditional melt-conveying models is that the polymer melt is assumed to be completely in a liquid phase. This assumption may be valid in the vicinity of the screw tip, while it must be critically readdressed for the remaining sections of the screw. Due to an incomplete melting process, solid and liquid materials may coexist side by side at the beginning of the melt-conveying zone. As a result, the polymer melt contains small portions of solid fragments that are trapped within the liquid phase. The influence of solid particles on the polymer melt flow is the topic of current research activities. Of particular interest are multiphase simulations of the liquid–solid mixture based on computational fluid dynamics (CFD), in which the solid material is represented by a highly viscous fluid.

A rather new but powerful method for deeper insight into liquid–solid multiphase flows requires the coupling of CFD and the discrete element method (DEM). The latter is widely accepted in the field of granular mechanics, and has already proven useful in the analysis of solids conveyed in single-screw extruders [162,163]. A coupled CFD/DEM approach can resolve both solid–solid and solid–liquid interactions and, hence, offers a promising method for predicting the flows of molten und unmolten materials. The usefulness of this modeling approach is not restricted to metering channels; rather, it offers great potential for a better understanding of all functional zones.

In addition to solving more complex mathematical models, there is an increasing need for further fast and accurate approximation methods. Removing the need for time-consuming and computationally expensive numerical methods, these techniques reduce calculation time, which is crucial in many time-critical applications. While existing equations were mainly designed to estimate the flow rate and viscous dissipation, further attention must be directed towards parameters such as the power consumption, temperature development, and mixing capability. Recent studies have demonstrated how analytical approximations constructed for screw channels of constant geometry can be implemented into a network-based approach to model the axial pressure and temperature development of both conventional and high-performance screws [140,142,164]. The development of new approximation methods will be promoted by the advent of data-based modeling and artificial intelligence. These techniques significantly extend the set of tools available to approximate large sets of experimental or simulation data (e.g., neural networks, decision trees, etc.).

## Figures and Tables

**Table 1:** Mathematical models of extruder flow of Newtonian fluids with temperature-independent viscosity.

No.	Flow	Equations	Boundary Conditions
1D_a	One-dimensional isothermal down-channel flow of a Newtonian fluid	(27) (η=const.)	(37)
1D_b	One-dimensional isothermal down-channel flow of a Newtonian fluid with wall effects	(22) (η=const.)	(37)
1D_c	One-dimensional isothermal cross-channel flow of a Newtonian fluid	(25) (η=const.)	(35), (42)
2D_a	Two-dimensional isothermal recirculating cross-channel flow of a Newtonian fluid	(19)–(21) (η=const.) $\left(\partial v_{x}/\partial x=\partial v_{y}/\partial x=0\right)$	(35), (36), (42)

**Table 2:** Mathematical models of a one-dimensional down-channel flow of a power-law fluid.

No.	Flow	Equations	Boundary Conditions
1D_d	One-dimensional isothermal down-channel flow of a power-law fluid	(16), (27), (31)	(37)
1D_e	One-dimensional isothermal down-channel flow of a power-law fluid with wall effects	$\left(16),\;(22),\;(23)\;(v_{x}=v_{y}=0\right)$	(37)
1D_f	One-dimensional non-isothermal down-channel flow of a power-law fluid	(16), (18), (27), (30), (31)	(37), (39), (40) or (41)

**Table 3:** Mathematical models of a two-dimensional flow of a power-law fluid in a screw channel of infinite width.

No.	Flow	Equations	Boundary Conditions
2D_b	Fully developed two-dimensional isothermal flow of a power-law fluid in a screw channel of infinite width	(16), (25)–(27), (29)	(35), (37), (42)
2D_c	Fully developed two-dimensional non-isothermal flow of a power-law fluid in a screw channel of infinite width	(16), (18), (25)–(29)	(35), (37), (39), (40) or (41), (42)
2D_d	Developing two-dimensional flow of a power-law fluid in a screw channel of infinite width	(16), (18), (25)–(27), (29), (34)	(35), (37), (39), (40) or (41), (42)

**Table 4:** Mathematical models of a three-dimensional flow of a power-law fluid in a screw channel of finite width.

No.	Flow	Equations	Boundary Conditions
3D_a	Fully developed three-dimensional isothermal flow of a power-law fluid in a screw channel of finite width	(16), (19)–(22), (23)	(35)–(37), (42)
3D_b	Fully developed three-dimensional non-isothermal flow of a power-law fluid in a screw channel of finite width	(16), (18), (19)–(23)	(35)–(37), (39), (40) or (41), (42)
3D_c	Developing three-dimensional flow of a power-law fluid in a screw channel of finite width	(16), (18), (19)–(22), (23), (33)	(35)–(37), (39), (40) or (41), (42)

**Table 5:** Overview of approximate methods based on correction factors or analytical regressions.

Year	Author	Target Variables	Flow Situation	Section
1969	Krüger	Flow rate	1D_d	Section 4.1
1981	Potente	Flow rate and power consumption	1D_d	Section 4.1
1981	Booy	Flow rate	2D_b	Section 4.2.1
1983	Potente	Flow rate and power consumption	2D_b	Section 4.2.1
1986	Rauwendaal	Flow rate	2D_b	Section 4.2.1
1995	Kim and Kwon	Flow rate	3D_a	Section 4.3.1
1996	Effen	Flow rate	2D_b	Section 4.2.1
1999	Obermann	Power consumption	3D_a	Section 4.3.1
2011	Spalding and Campbell	Flow rate	3D_a	Section 4.3.1
2017	Pachner et al.	Flow rate	2D_b	Section 4.2.1
2017	Marschik et al.	Flow rate	3D_a	Section 4.3.1
2018	Roland and Miethlinger	Viscous dissipation	1D_d and 2D_b	Section 4.1/Section 4.2.1
2019	Roland	Flow rate	1D_d	Section 4.1
2019	Roland et al.	Flow rate and viscous dissipation	2D_b	Section 4.2.1
2019	Roland et al.	Viscous dissipation	3D_a	Section 4.3.1

**Table 6:** Overview of the three-, two-, and one-dimensional modeling approaches compared.

No.	Model	Literature	Flow Situation	Section	Modifications
1	Marschik et al.	[116]	3D_a	Section 4.3.1	-
2	Rauwendaal	[125]	2D_b	Section 4.2.1	Shape factors
3	Effen	[130]	2D_b	Section 4.2.1	Shape factors
4	Roland et al.	[106]	2D_b	Section 4.2.1	Shape factors
5	Roland	[136]	1D_d	Section 4.1	Shape factors
6	Newtonian pumping model	[1]	1D_b	Section 3.1	Shape factors

**Table 7:** Overview of the parameter ranges of the datasets and the models used in each case.

**No.**	n	$t/D_{b}\;$	$h/w_{b}\;$	$\Pi_{p,z}\;$	$\Pi_{V}$	Models
Dataset 1	0.2–1.0	0.6–2.0	0.05–0.5	−1.0–var.	-	1, 4, 5, 6
Dataset 2	0.2–1.0	0.8–2.0	0.05–0.5	−1.0–var.	0.1–2.0	1, 3, 4, 5, 6
Dataset 3	0.2–1.0	0.84–1.46	0.05–0.5	−1.0–var.	0.1–2.0	1–6

**Table 8:** Quality measures of the approximations: mean absolute error (MAE ) and coefficient of determination ($R^{2}$ ).

No.	Model		Dataset 1	Dataset 2	Dataset 3
1	Marschik et al.	MAE	0.00719	0.00673	0.00555
		$R^{2}$	0.99973	0.99967	0.99980
2	Rauwendaal	MAE	-	-	0.05290
		$R^{2}$	-	-	0.97291
3	Effen	MAE	-	0.10934	0.18908
		$R^{2}$	-	0.02351	−1.07304
4	Roland et al.	MAE	0.02681	0.02465	0.02363
		$R^{2}$	0.99433	0.99244	0.99344
5	Roland	MAE	0.11079	0.09800	0.09974
		$R^{2}$	0.90623	0.90105	0.99344
6	Newtonian pumping model	MAE	0.17595	0.14418	0.14149
		$R^{2}$	0.83683	0.84890	0.85426

## Data Availability

The data presented in this study are available on request from the corresponding author.

## Nomenclature

$a_{t}\;$	Temperature shift factor	v	Characteristic velocity
$c_{p}$	Specific heat capacity (constant pressure)	$v_{b}$	Barrel velocity
$c_{v}$	Specific heat capacity (constant volume)	$v_{b,x}$	Barrel velocity in the x-direction
Br	Brinkman number	$v_{b,z}$	Barrel velocity in the z-direction
$D_{b}$	Barrel diameter	$v_{i}$	Velocities
$D_{s}$	Screw core diameter	v	Velocity vector
D	Rate-of-deformation tensor	$\dot{V}$	Volume flow rate
e	Flight width	${\dot{V}}_{d}$	Drag flow rate
f	Degree of filling	${\dot{V}}_{p}$	Pressure flow rate
$f_{L}$	Correction factor for leakage flow	$w_{b}$	Channel width at barrel surface
$F_{d}$	Shape factor (drag flow)	$w_{b,f}$	Width of filled channel
$F_{d,f}$	Shape factor (drag flow), partially filled	$w_{b,uf}$	Width of unfilled channel
$F_{p}$	Shape factor (pressure flow)	x	Cross-channel coordinate
g	Gravity vector	y	Up-channel coordinate
h	Channel depth	z	Down-channel coordinate
i	Number of screw flights	Z	Unwound length
K	Consistency	α	Temperature coefficient
L	Characteristic length	δ	Flight clearance
L	Velocity gradient tensor	$\dot{\gamma }$	Shear rate
MAE	Mean absolute error	η	Viscosity
n	Power-law index	$\eta_{f}$	Viscosity in the flight clearance
N	Screw speed	λ	Heat conductivity
$R^{2}$	Coefficient of determination	$v_{d}$	Dimensionless velocity (drag flow)
Re	Reynolds number	$v_{i}$	Dimensionless velocities
p	Pressure	$v_{p}$	Dimensionless velocity (pressure flow)
$P_{Drive}$	Drive power	$\Pi_{p,i}^{}$	Dimensionless pressure gradients
Pe	Péclet number	$\Pi_{Q}^{}$	Dimensionless dissipation
${\dot{q}}_{Diss}$	Viscous dissipation	$\Pi_{V}^{}$	Dimensionless flow rate
t	Screw pitch	ρ	Density
T	Temperature	$\tau_{ij}$	Shear stresses
$T_{0}$	Reference temperature	τ	Stress tensor
$T_{b}$	Barrel temperature	$\varphi_{b}$	Pitch angle
$T_{s}$	Screw temperature		
